# An MCEM Framework for Drug Safety Signal Detection and Combination from Heterogeneous Real World Evidence

**DOI:** 10.1038/s41598-018-19979-7

**Published:** 2018-01-29

**Authors:** Cao Xiao, Ying Li, Inci M. Baytas, Jiayu Zhou, Fei Wang

**Affiliations:** 1 AI for Healthcare, IBM Research,, Cambridge, USA; 2 Center for Computational Health, IBM T. J. Watson Research Center, Yorktown Heights, USA; 3 Department of Computer Science and Engineering, Michigan State University, East Lansing, USA; 4 Department of Healthcare Policy and Research, Weill Cornell Medical School, Cornell University, New York, USA

## Abstract

Delayed drug safety insights can impact patients, pharmaceutical companies, and the whole society. Post-market drug safety surveillance plays a critical role in providing drug safety insights, where real world evidence such as spontaneous reporting systems (SRS) and a series of disproportional analysis serve as a cornerstone of proactive and predictive drug safety surveillance. However, they still face several challenges including concomitant drugs confounders, rare adverse drug reaction (ADR) detection, data bias, and the under-reporting issue. In this paper, we are developing a new framework that detects improved drug safety signals from multiple data sources via Monte Carlo Expectation-Maximization (MCEM) and signal combination. In MCEM procedure, we propose a new sampling approach to generate more accurate SRS signals for each ADR through iteratively down-weighting their associations with irrelevant drugs in case reports. While in signal combination step, we adopt Bayesian hierarchical model and propose a new summary statistic such that SRS signals can be combined with signals derived from other observational health data allowing for related signals to borrow statistical support with adjustment of data reliability. They combined effectively alleviate the concomitant confounders, data bias, rare ADR and under-reporting issues. Experimental results demonstrated the effectiveness and usefulness of the proposed framework.

## Introduction

Adverse drug reactions (ADR) cause a global and substantial burden accounting for considerable mortality and morbidity, as well as extra costs due to increased hospitalization, prescription cascades, and other ADR consequences^1^. Unanticipated ADRs may occur after a drug has been approved due to its use or prolonged use on large, diverse populations. Therefore, the post-marketing drug safety surveillance, also referred to as pharmacovigilance^2^, has become an essential component to monitor unanticipated ADRs, generate more complete drug safety profiles, and assist governmental drug administration agencies to take actions against these risks^3^.

To facilitate pharmacovigilance research, Real World Evidence (RWE) data that are generated from real world practice to reflect actual patient experience become increasingly important. The drug-related RWE mainly comprises of two types of data: the reports from Spontaneous Reporting Systems (SRS) submitted by pharmaceutical companies, healthcare professionals and consumers, as well as the Observational Health Data (OHD) including electronic health records, patient registries, and administrative claims. Among the two types of RWE, the SRS has served as a cornerstone for post-marketing drug surveillance, and the FDA Adverse Event Reporting System (FAERS) is one of the most prominent SRSs. The FAERS consists of a collection of case reports, each of which includes a few adverse events associated with the administration of several drugs. These case reports have provided rich evidence to assist the early identification of drug safety signals and generate hypothesis for further confirmatory investigations, sometimes regulatory warnings and changes of product information^4^, and even withdrawals of marketing authorizations^5^. For example, signals indicating the strong associations between cerivastatin and rhabdomyolysis has led to various regulatory decisions between 1999 and 2001^6^; signals indicating associations between antihistamine Seldane and fatal heart rhythm disturbance caused the drug to be pulled out of market^7^.

Due to the valuable information SRS data can provide, mining drug safety signals from SRS has become a highly active research area^8–10^. During the past decades, a series of Disproportionality Analysis (DPA) methods were developed to automatically detect drug safety signals from SRS. The basic idea of disproportionality analysis lies on the assumption that combinations of a drug and a clinical event that are disproportionately highly represented in the database may indicate an important risk signal based upon a difference from the background frequency. The most widely cited DPA measurements are the Relative Reporting Ratio (RRR), Proportional Reporting Rate (PRR) and Reporting Odds Ratio (ROR)^11,12^. However, they all suffer from the sampling variance issue^13,14^. To address this issue, two Bayesian methods, Multi-item Gamma Poisson Shrinker (MGPS)^15^ and Bayesian Confidence Propagation Neural Network (BCPNN)^16^ were proposed. Both approaches handle sampling variance by shrinking relative reporting ratio or information component towards a prior when less data concerning the drug-ADR pair is available^15,16^. Particularly, MGPS is considered reliable and in routine use by FDA. As for OHD data, recent years it becomes an emerging promising resource of pharmacovigilance^17–20^. Also, since different types of RWE data are collected with various designs and approaches, weaving together multiple RWE sources could further improve the quality and efficiency of drug safety detections. Previously, a Bayesian hierarchical model^10^ was proposed to combine signals from SRS and OHD, while ranking methods were applied to combine signals from SRS and literature^17^. However, the aforementioned signal detection and combination methods still have large potentials of improvement. We identify several challenges as below.

1. *Challenges of Explicit Handling Concomitant Confounders in SRS Data*: Concomitant drugs partially inherit each other’s associations^8^. When only one drug is the cause of a particular ADR, the concomitant drugs become confounders. Such issue has rarely been explicitly handled by existing works.
2. *Challenges of Data Related Issues in Single RWE Data*: There are several data related issues for a single source of RWE data, including (i) ADR cases in the SRS are known to be under-reported^14^, (ii) Although RWE data is a potentially transformative force in healthcare, these data still do not yet suffice to fully overcome the fundamental issues of data quality and bias^21,22^, and (iii) True drug-ADR associations are often considered rare events compared with large amounts of case reports.In order to capture rare but critical ADRs, the DPA methods (e.g. MGPS) would suffer from high false positive rates^16^.
  In this work, we propose to alleviate these issues with the following two steps.
  1. *Monte-Carlo Expectation Maximization Step (MCEM)*: To filter out concomitant confounders in each case report, we propose a new Monte Carlo sampling procedure that could assign each ADR with its major associated drug determined by drugs’ contribution to that ADR (e.g. measured by normalized MGPS) in the case report. This is achieved via an iterative procedure: we start with calculating MGPS scores for all drug-ADR pairs based on all reports, then for each report we normalize the MGPS scores across drugs in the report to obtain drugs’ probability proportional to their MGPS scores related to the given ADR. Next we perform iterative Monte Carlo sampling to sample one drug based on such probability. In each iteration, the sampled drug is added to the report saved throughout previous iterations and MGPS scores are re-calculated for the current report. With such a procedure, we can generate more accurate SRS signals for each ADR through iteratively down-weighting their associations with irrelevant drugs in case reports.
  2. *Signal Combination Step*: To alleviate the issues of under-reporting, data bias, and rare ADR detections from a single RWE source, we adopt an empirical Bayesian approach to combine different signals generated from multiple and different types of RWE sources. It not only generates improve drug safety signals, but also has a smoothing effect to prevent performance degradation due to anomalies and artifacts in some data source. Moreover, to account for the data quality, we propose a new summary statistic that takes a pooling strategy to put more emphasis on more reliable data sources.

## Materials and Methods

### Data Description

#### FDA Adverse Event Reporting System (FAERS)

The SRS data used in this study is the FAERS data from 2007 to 2014, including 437,317 reports per year on average. To preprocess the data, Banda *et al*. has curated a cleaned and standardized version of FAERS with duplicate case records removed, mapping drug names to RxNorm concepts and ADR outcomes to MedDRA concepts^23^ (http://datadryad.org/resource/doi:10.5061/dryad.8q0s4). Based on this dataset, we further mapped relevant MedDRA concepts to four ADRs of interest in the gold standard supplied by OMOP, including acute myocardial infarction (AMI), acute liver injury (ALI), acute renal failure (ARF), and upper GI bleeding (UGB).

#### Canada’s Spontaneous Reporting System (MedEffect)

To test the robustness of the MCEM approach, we further evaluated on another independent adverse event database, Canada’s MedEffect database (https://www.canada.ca/en/health-canada/services/drugs-health-products/medeffect-canada.html). MedEffect is a Canadian version of the FAERS and contains ~250,000 adverse event reports from 2004 to 2014. To preprocess the data, we also mapped drug names to RxNorm concepts and ADR outcomes to MedDRA concepts. Based on this dataset, we further mapped relevant MedDRA concepts to three ADRs of interest in the gold standard supplied by OMOP, including AMI, ALI, and UGB. Unlike FAERS, ARF was not included here since the preferred MedDRA term of ARF (term ID: 10038436) did not appear in MedEffect database. The final OMOP reference standard for MedEffect database involves three ADRs of interest, 61 true positive cases and 15 true negative cases.

#### Observational Healthcare Database (OHD)

For the signal combination step, the OHD used in this work is from Truven Health Analytics Commercial Claims and Encounters (CCAE) (http://truvenhealth.com/markets/life-sciences/products/data-tools/marketscan-databases), which represents the privately insured population and captures administrative claims with patient-level de-identified data from inpatient and outpatient visits, and pharmacy dispensing claims from the outpatient setting^12^. The database used in the study involves 46.5 million patients from 2003 to 2009, among them, 49% are male and the mean age is 31.4. In total, it includes 1,030.6 million national drug codes from pharmacy dispensing claims and 1,257.5 million ICD9 codes from inpatient and outpatient claims.

### The Signal Detection and Combination Framework

In this section, we propose the signal detection and combination framework. The objective is to generate a grand risk score for each drug-ADR pair, where confounders will be filtered using MCEM and the signals will be enhanced via combining multiple data with a Bayesian hierarchical model.

**Figure 1:**
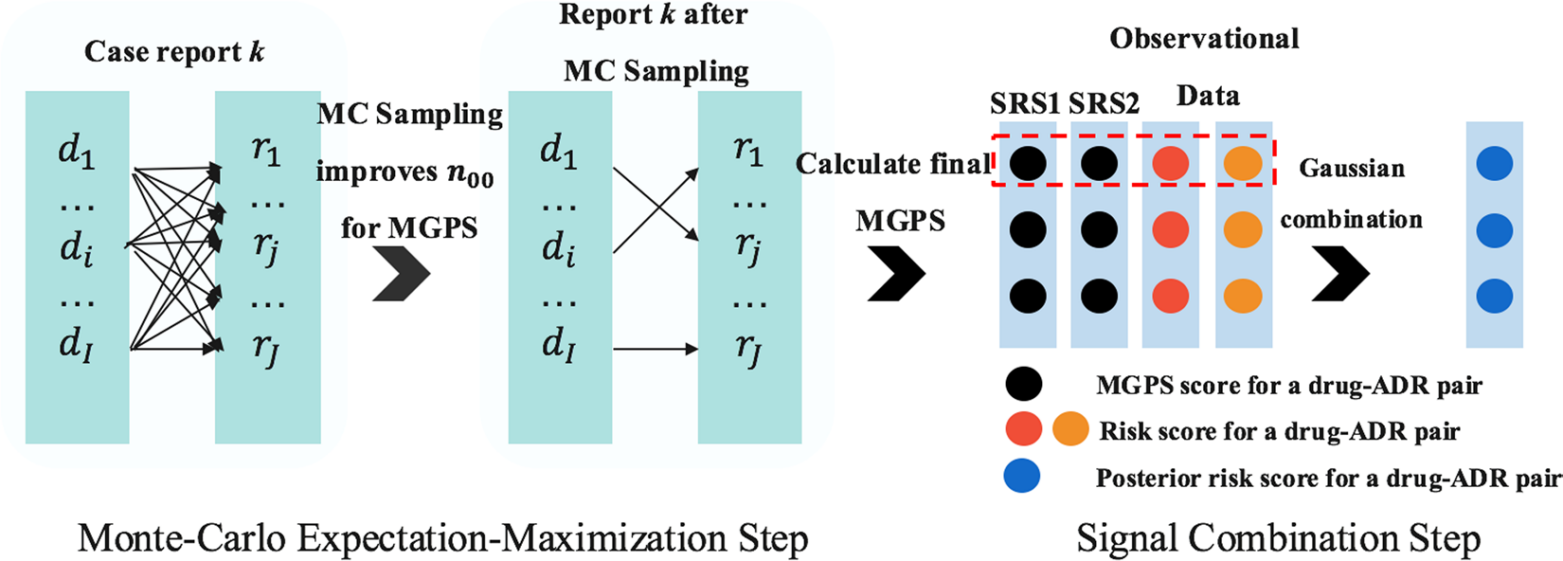
The proposed signal detection and combination framework: For a particular case report, given a set of drugs and a set of ADRs, MCEM procedure is used to filter out the concomitant drug confounders to associate each ADR with one major drug. To further enhance the signal strength, an empirical Bayesian based signal combination approach is used to combine signals from OHD data with signals from SRS case reports.

The overall framework is illustrated in Fig. 1, where *D* = {*d*_1_, …, *d*_*I*_} represents a set of drugs given for a case report and *R* = {*r*_1_, …, *r*_*j*_} denotes a set of ADRs. There are multiple co-occurrences between one ADR and several drugs as shown in Fig. 1. However, it is assumed that for each ADR in each report there exists a single drug as its major cause while the other drugs are confounders that need to be filtered out. This assumption consequently leads to an improved drug-ADR co-occurence count used in MGPS computation, and thus yields more accurate results. Improved drug-ADR counts are obtained by the MC sampling procedure, where for each ADR, drugs are sampled with a probability proportional to its MGPS score, which effectively gives more emphasis on the drugs with higher probability to be associated with the corresponding ADR. After the enhanced counts are obtained, we can compute final MGPS scores for each drug-ADR pair. In the signal detection step, we combine signal scores from SRS with their counterpart safety signal scores from other OHD (e.g. claims) data via an empirical Bayesian model that enables related signals to borrow statistical support from one another with adjustment of data reliability. In the following sections, we discuss each step of the proposed framework.

#### The Multi-item Gamma Poisson Shrinker (MGPS) Method

The spontaneous reporting systems (SRS) used in this study are of the following structure: each dataset consists of numerous records and each record contains a set of patient reported ADR instances along with drugs that are suspected to cause the ADRs. The MGPS method^15^ is a leading disproportionality analysis method for detecting potential drug safety signals from the SRS. It focuses on low-dimensional projections of the data, specifically 2-dimensional contingency tables as illustrated in Table 1. The MGPS method is computed as follows: let *n*_00_(*i*, *j*) denote the *N*_*ij*_ entry for the number of events regarding the *i*th drug and the *j*th ADR. Assume each observation of *N*_*ij*_ is drawn from a Poisson distribution with an unknown mean *μ*_*ij*_, then the theoretical value of relative risk between the *i*th drug and the *j*th ADR, *λ*_*ij*_, can be computed using Eq. 1.

1 $${\lambda }_{ij}=\frac{{\mu }_{ij}}{{E}_{ij}}=\frac{{\mu }_{ij}}{[{n}_{0+}(i,j){n}_{+1}(i,j)/{n}_{++}(i,j)]}$$

where $${n}_{0+}(i,j)={n}_{00}(i,j)+{n}_{01}(i,j)$$, $${n}_{+1}(i,j)={n}_{01}(i,j)+{n}_{11}(i,j)$$, and $${n}_{++}(i,j)={n}_{00}(i,j)+{n}_{11}(i,j)+$$$${n}_{10}(i,j)+{n}_{01}(i,j)$$. Here we regard the geometric mean of the posterior distribution for each *λ*_*ij*_ as the MGPS scores. And *λ*_*ij*_ is assumed to arise from a particular 5-parameter prior distribution, namely a mixture of two gamma distributions as given in Eq. 2.

2 $${\lambda }_{ij}\sim wGa({\alpha }_{1},{\beta }_{1})+\mathrm{(1}-w)Ga({\alpha }_{2},{\beta }_{2})$$

where *Ga* indicates the Gamma distribution, and *α*_1_, *β*_1_ and *α*_2_, *β*_2_ are their hyperparameters.

**Table 1:** 2 × 2 Contingency Table.

	Report with ADR	Report without ADR
Report with Drug	*n*_00_	*n*_01_
Report without Drug	*n*_10_	*n*_11_

Then then posterior distribution of *λ*_*ij*_ is iteratively fitted based on the observations of data under the Bayesian framework as is given by Eq. 3.

3 $${\lambda }_{ij}|{N}_{ij}={n}_{00}\sim wGa({\alpha }_{1}+{n}_{00},{\beta }_{1}+{E}_{ij})+\mathrm{(1}-w)Ga({\alpha }_{2}+{n}_{00},{\beta }_{2}+{E}_{ij})$$

Based on the posterior, the criteria for generating a signal is initially proposed by DuMouchel^15^ as ranking drug-ADR pairs by their posterior expectation of log_2_(*λ*_*ij*_), which can be expressed by the following Eq. 4.

4 $$E[{\mathrm{log}}_{2}({\lambda }_{ij}|{N}_{ij}={n}_{00})]=\frac{{Q}_{n}[\psi ({\alpha }_{1}+{n}_{00})-\,\mathrm{ln}({\beta }_{1}+{E}_{ij})]+\mathrm{(1}-{Q}_{n})[\psi ({\alpha }_{2}+{n}_{00})-\,\mathrm{ln}({\beta }_{2}+{E}_{ij})]}{\mathrm{ln}\,2}$$

where ψ is the diagamma function and *Q*_*n*_ can be computed from Eqs 5 and 6.

5 $${Q}_{n}=\frac{wf({n}_{00};{\alpha }_{1},{\beta }_{1},{E}_{ij})}{wf({n}_{00};{\alpha }_{1},{\beta }_{1},{E}_{ij})+\mathrm{(1}-w)f({n}_{00};{\alpha }_{2},{\beta }_{2},{E}_{ij})}$$

6 $$f({n}_{00};{\alpha }_{1},{\beta }_{1},{E}_{ij})={\mathrm{(1}+\beta /E)}^{-{n}_{00}}{\mathrm{(1}+E/\beta )}^{-\alpha }\frac{{\rm{\Gamma }}(\alpha +{n}_{00})}{{\rm{\Gamma }}(\alpha ){n}_{00}}\mathrm{.}$$

To conclude, the adoption of MGPS method not only provides a shrinkage estimate of relative risk to address the sampling variance issue, but also works efficiently on large-scale data. It is considered an important improvement, and thus becomes the most widely used methods that is in routine use by regulators (e.g. FDA) and pharmaceutical manufacturers worldwide. However, the confounders induced by concomitant drugs still remain and would cause inaccurate detections. In the following, we introduce an MCEM framework to further address such confounding issues in MGPS.

#### Monte Carlo Sampling Step

To alleviate the confounding effects induced by concomitant drugs, we borrow the idea of discrete choice models^24^ and try to filter out these confounders by assuming for each case report, each ADR has at most one major associated drug. Although some ADRs could be induced by drug combinations (i.e. drug drug interactions (DDI)), previous study confirmed the DDI incidences could be quite rare among reports involving at least two drugs in SRS databases^25^. Therefore, for most cases the “one major drug” assumption holds, though this procedure could be extended to multiple drugs. Based on such assumption, we propose the following MCEM procedure.

Here is a practical guide for the MCEM procedure. In an MCEM procedure, the maximizer of the posterior probability is approximated with sampled data in the E-step and the value of the maximizer is optimized in the M-step. In our case, we first compute MGPS scores for all drug-ADR pairs across all reports in the SRS system. Then, for each ADR in each report, we normalize the MGPS scores across only drugs in this report to obtain these drugs’ contribution ratio proportional to their MGPS scores related to the given ADR. And these contribution ratio will be used as the sampling probabilities and we will draw from multinomial distribution to assign the major drug to the target ADR in the next step. In the next step, we perform iterative Monte Carlo sampling to sample one drug based on the aforementioned probabilities. In each iteration, the sampled drug is added to the report saved throughout these iterations and MGPS scores are re-calculated for the current states over all reports. In the next iteration, updated MGPS scores for all drug-ADR pairs are used and we iterate the process until the difference between the optimal values of the maximizer in consecutive iterations is less than a heuristic threshold (e.g. 10^−3^, 10^−5^). Last we compute final MGPS scores for all drug-ADR pairs. Note that a general description of MCEM algorithm is provided in Appendix A1. and an algorithmn version is in Procedure A2.

### Significance

The Monte Carlo sampling procedure assigns the major drug for each ADR ranked by their contribution to the risk score. After iterations, the procedure will down-weight irrelevant causes (e.g. drugs) for each ADR and thus generate improved the count of co-occurrence of a drug and ADR pair for all drug-ADR pairs.

#### Signal Combination Step

In the signal combination step, we employ an empirical Bayesian strategy to combine drug safety signals obtained from multiple data sources. We formulate the signal combination as a Bayesian hierarchical model that assumes signals are independently and identically distributed with shared hyper-parameters. Index drug-ADR pair (*i*, *j*) with $l\in \mathrm{\{1,}\cdots ,L\}$, and *y*_*lk*_ as the quantified relationship between *l*-th drug-ADR pair from *k*-th $(k\in \mathrm{\{1,}\cdots ,K\})$ data source. In addition, we define ${\sigma }_{lk}^{2}=Var({y}_{lk})$ as the observed variance of *y*_*lk*_. Then the objective becomes to estimate the combined score *ϕ*_*l*_ for the *l*-th drug-ADR pair with $Y=\{{y}_{lk}\}$ and $S=\{{\sigma }_{lk}^{2}\}$. Here we follow the idea in Harpez *et al*^10^. and assume the observed scores ${y}_{l1},\cdots ,{y}_{lK}$ follow a Gaussian process centered around *ϕ*_*l*_, where *ϕ*_*l*_ follows a Gaussian distribution centered around grand prior mean *θ* which allows related signals to share statistical properties. These relations are given by the following distributions defined in Eqs 7 and 8:

7 $$p({y}^{(l)}|{\varphi }_{l},\theta )\sim N({\varphi }_{l},{\sigma }_{l}^{2})$$

8 $$p({\varphi }_{l}|\theta )\sim N(\theta ,{\tau }^{2}\mathrm{).}$$

And the signal combination is computed as the estimate of ${\varphi }_{l}$ as given by Eq. 9:

9 $${\hat{\varphi }}_{l}={c}_{l}{y}^{(l)}+\mathrm{(1}-{c}_{l})\theta $$

where ${y}^{(l)}$ is a summary statistic that is meant to summarize the information (signal scores) provided by each data source for a given drug-ADR association. The summary statistic ${y}^{(l)}$ is for approximating the joint density of the scores and *ϕ*, which is used to obtain the posterior distribution of *ϕ* and ${c}_{l}=\frac{{\tau }^{2}}{{\tau }^{2}+{\sigma }_{l}^{2}}$. In addition, we also follow the notation as in Harpez *et al*^10^. to denote ${\hat{\varphi }}_{l}$ as the mean of the posterior distribution of *ϕ*_*l*_ given *θ*, ${\tau }^{2}$ and the scores.

Here, ${\tau }^{2}$ and *θ* are estimated via expectation maximization (EM) with the independently distributed observations ${y}^{(l)}$ conditioned on *ϕ*_*l*_. Thus, we perform maximum likelihood estimation of the hyper-parameters using the posterior distribution of *ϕ* given the scores and their variances in each iteration. Note that, in Harpez *et al*.^10^, summary statistic ${y}^{(l)}$ is defined assuming signals from different sources have approximately the same scale. While in our work, we define ${y}^{(l)}$ in a way that the signal sources with less uncertainty would be emphasized more. To be specific, ${y}^{(l)}$ is calculated as a weighted average of the scores obtained by the same source first, then average of variances of individual scores is used as a weighting coefficient to combine different data sources. The formula is given below by Eqs 10 and 11.

10 $${y}^{(l)}=\sum _{k\mathrm{=1}}^{K}\{\frac{1}{\sum _{m=1}^{{N}_{k}}(\sum _{l=1}^{L}{\sigma }_{lm}^{2}/{N}_{k})}\cdot \frac{\sum _{m=1}^{{N}_{k}}(\sum _{k}{y}_{lm}/{\sigma }_{lm}^{2})}{\sum _{m=1}^{{N}_{k}}(\sum _{k}1/{\sigma }_{lm}^{2})}\}$$

11 $${\sigma }_{l}^{2}=Var({y}^{(l)})$$

where *N*_*k*_ is the number of signals from the *k*-th source.

### Significance

The proposed signal combination step can be considered a pooling strategy: For the same drug-ADR pair, if the average uncertainty of one data source is high overall, then signal combination will put more weights on other data sources with less uncertainty. This approach also provides a smoothing effect since each drug-ADR pair has safety scores (e.g. MGPS or RR) from several sources, combining signals from multiple sources will prevent the performance of signal detection from degradation when there is artifact or data anomaly in one or more sources.

#### Evaluation Method

We evaluate the proposed methodology using a gold standard created and validated by the Observational Medical Outcomes Partnership (OMOP), including 380 positive and negative test cases (drug-outcome pairs) in total. Positive test cases are true ADR associations asserted from drug labeling (mention of an outcome as an adverse reaction) and literature. While negative test cases are associations that lack this level of evidence in their labeling or literature. The entire gold standard includes 181 drugs and is divided into four sets, each associated with a unique outcome including acute myocardial infarction, acute renal failure, acute liver injury, and upper gastrointestinal bleeding, which represent four of the most significant and actively monitored drug safety outcomes^26^.

In performance comparison, we use the area under the ROC curve (AUC) as the evaluation metric. We are also interested in performance at fixed thresholds and levels. For example, the lower bound of the 90% confidence interval for the Empiric Bayes Geometric Mean. denoted as EB05, is greater than 2 (i.e. EB05 > 2). The EB05 > 2 is a popular fixed definition of drug safety signal^11,23^.

### Data Availability

The FAERS datasets used in the current study is available in http://datadryad.org/resource/doi:10.5061/dryad.8q0s4. The proposed framework implemented using R, along with results generated in the study are available in the mcem-drug-safety repository, https://github.com/danicaxiao/mcem-drug-safety.

## Results

### Performance evaluation on Real-World Data

#### Performance for the MCEM Step

In this part, we evaluate the performance of the MCEM step. Average AUCs based on ADRs of interest are reported in Tables 2 and 3. Results show that the proposed MCEM step generates more accurate signals, and thus brings significant AUC gains for overall data, as well as most ADRs. For the ALI cases, the proposed method did not work well, which could be due to the unusual data distribution of ALI, e.g. more cases than controls. Typically ADRs are rare events, where there are many more controls than cases. In the ALI case, our method could under-estimate the risk significance of some true positive cases when there are more positive cases in the data.

**Table 2:** Comparison of the standard MGPS score and MCEM MGPS score based on ADRs of interest in FAERS.

SRS Dataset	Average AUC (MGPS)	Average AUC (MCEM MGPS)
All ADRs	0.6787	**0.7225**
Acute Myocardial Infarction (AMI)	0.5834	**0.6109**
Acute Liver Injury(ALI)	**0.6659**	0.6512
Acute Renal Failure (ARF)	0.6926	**0.8243**
Upper GI Bleeding (UGB)	0.6610	**0.7660**

**Table 3:** Comparison of the standard MGPS score and MCEM MGPS score based on ADRs of interest in MedEffect.

SRS Dataset	Average AUC (MGPS)	Average AUC (MCEM MGPS)
All ADRs	0.7366	**0.7683**
Acute Myocardial Infarction (AMI)	0.7500	**0.7812**
Acute Liver Injury(ALI)	**0.6829**	0.4756
Upper GI Bleeding (UGB)	0.7500	**0.7870**

Also we evaluate the performance by the reporting ending years in Table 4. From the results, we observe the following trend: the more years of reports we collect, the better prediction performance we can achieve with the proposed MCEM procedure. However, MGPS calculation based on original reports could not guarantee this. Comparing between the proposed method and the original approach, we can see that the proposed MCEM step generates more accurate signals for every reporting year.

**Table 4:** Comparison of the standard MGPS score and MCEM MGPS score by reporting ending years.

SRS Dataset	AUC (MGPS)	AUC (MCEM MGPS)
As of FAERS 2007	0.6994	**0.7265**
As of FAERS 2008	0.6936	**0.7067**
As of FAERS 2009	0.6889	**0.7096**
As of FAERS 2010	0.6853	**0.7282**
As of FAERS 2011	0.6868	**0.7353**
As of FAERS 2012	0.6907	**0.7146**
As of FAERS 2013	0.7091	**0.7261**
As of FAERS 2014	0.7012	**0.7385**

**Figure 2:**
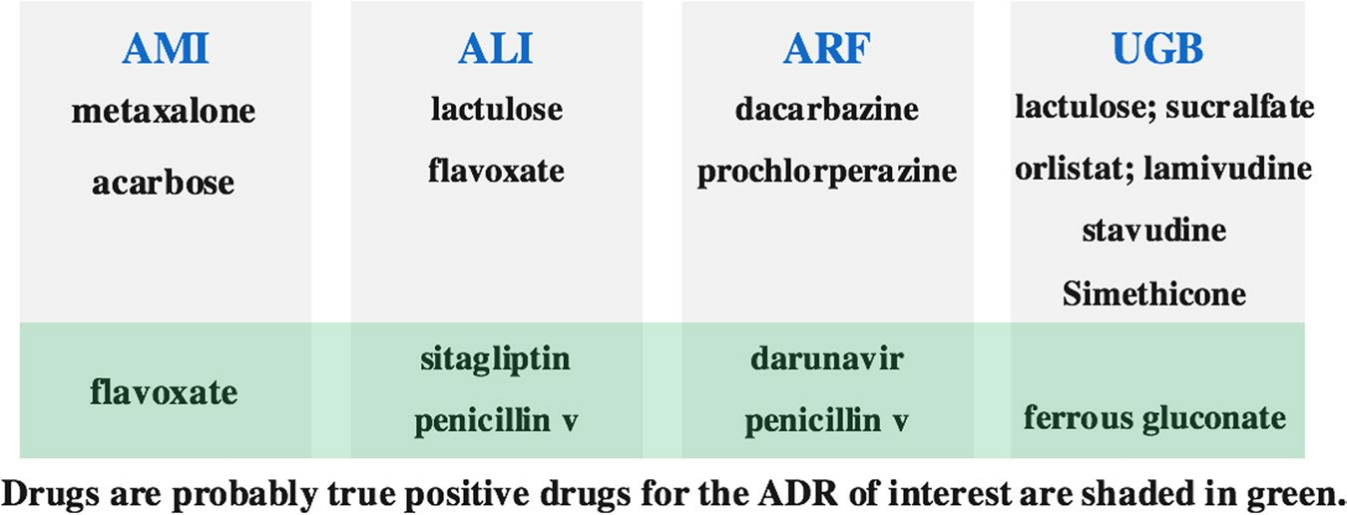
False positive signals detected by MCEM.

In addition to performance evaluation, we also examine false positive predictions of the proposed method. We have considered 102 drugs for AMI, 115 drugs for ALI, 88 drugs for ARF, 91 drugs for UGB. The drugs were selected according to the strict criteria in^27^. We report the precision for both proposed method and baseline methods in Table A4. Here our fucos is on showing some of false positive signals may be the true signals are listed in Fig. 2. The drugs are selected based on the criteria of EB05 > 2 for each year. Among the four ADRs, the UGB has the most false positive drugs. The reason could be related to what has been discussed in Hauben *et al*.^28^, where a study discovered that 40 ‘negative controls’ in the reference standard might have been misclassified. According to the evidence^28^, six drug-ADR pairs that are identified as false positive cases are in fact true positive signals (shaded in green color).

#### Performance of Signal Combination

For the signal combination step, we consider the following inputs: MGPS scores computed from the MCEM step, and the safety scores calculated from OHD. For baselines, we also consider combining original MGPS scores with safety scores calculated from OHD. For the combination method, rank aggregation^29^ and the empirical Bayesian (EB) signal combination method^10^ are reported as baselines. Signal combination results are given in Table 5. In experiments, the signal combinations are applied on the common drug-ADR pairs obtained from all sources.

**Table 5:** Performance comparison of risk signal combination methods.

	AUCs of Different Combination Methods		
	Rank Aggregation	Empirical Bayesian in^10^	Proposed Framework
**Signals**			
Raw MGPS + OHD	0.5176	0.7540	X
MCEM MGPS + OHD	0.6290	0.7584	**0.8606**

From Table 5, we can see that the proposed framework outperforms all baseline methods significantly. The bad performance of rank aggregation is due to the high reliability variance across different years (e.g. AUC ranging from 0.6489 to 0.7614), as well as the label imbalance for each year (e.g. much more cases than controls). Since the rank aggregation method is optimized to reach a consensus among all base rankers, drug-ADR pairs that show signals in some years will be compromised due to lack of signals in other years. Thus, many cases would be incorrectly labeled as controls, which consequently degrades the combined list such that the final results are generally worse than individual rankers. Another baseline EB, which was proposed in Harpez *et al*.^10^, is a state-of-the-art method. Since it takes weighted average without considering the reliability of different data sources, the performance is not as good as the proposed method.

### Case Studies

#### Sampling mechanism and its effect on the SRS

The sampling step is designed to select the major drug from multiple drugs for a particular ADR within each case report. Note that case reports mention multiple drugs have accounted for 48% of overall case reports between the years of 2007 and 2014. For the reports that mention multiple drugs, we compare the agreement between the drugs assigned by the sampling step and primary suspected drugs assigned by reporters. On average, 34.4% of assignments were the same by two mechanisms.

#### Early detection of true positive signals

**Figure 3:**
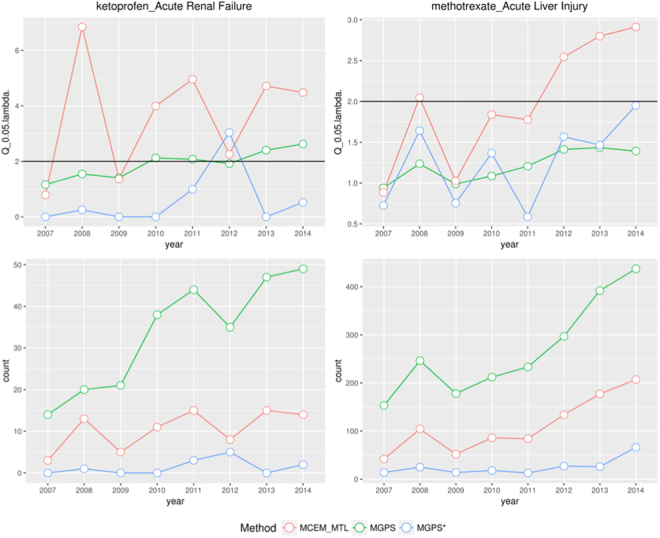
Comparison of early detection of true positive signals: ketoprofen causing acute kidney injury (left), and methotrexate causing acute liver injury (right).

As shown in Fig. 3 (upper-left), by using the industrial threshold EB05 > 2, the proposed method was able to detect the ketoprofen-ARF (i.e. acute kidney injury) signal as early as of 2008. As a contrast, the traditional MGPS based on full data set was not able to detect this signal, and the MGPS based on primary suspected data set was only able to detect it until 2014. Figure 3 (lower-left) shows that there was only one case report specified by the reporter that ketoprofen was the causal drug for acute failure in 2008, however, the proposed method identified that in fact in 13 case reports acute renal failure was caused by ketoprofen in 2008. We further evaluated these 12 discordant case reports manually, whereas celecoxib, zoledronic acid, acetaminophen/tramadol oral tablet, capecitabine, dipyridamole, ibuprofen, simvastatin, and valsartan were mentioned by the reporters to be the causes of acute renal failure, respectively.

Another example in Fig. 3 (upper-right) is the detection of methotrexate inducing ALI. The MCEM method can identify this signal in 2008, 2012, 2013 and 2014 respectively while neither MGPS nor MGPS* (that indicates the MGPS scores computed based on primary drugs identified by patients themselves) can detect this signal. In 2008, the reporters mentioned the methotrexate as the primary suspected reason for ALI in 25 case reports, while MCEM method selected methotrexate as the major drug for ALI in 104 case reports. Among 79 discordant case reports, etanercept (36 times), adalimumab (13 times) and busulfan (8 times) were the most frequently mentioned as primary suspected drugs. In general, 16 true positive signals were only detected by the MCEM, two true positive signals were only detected by MGPS* and neither of true positive signals was detected by MGPS alone.

#### ADR evidence strengthen through signal combination

**Figure 4:**
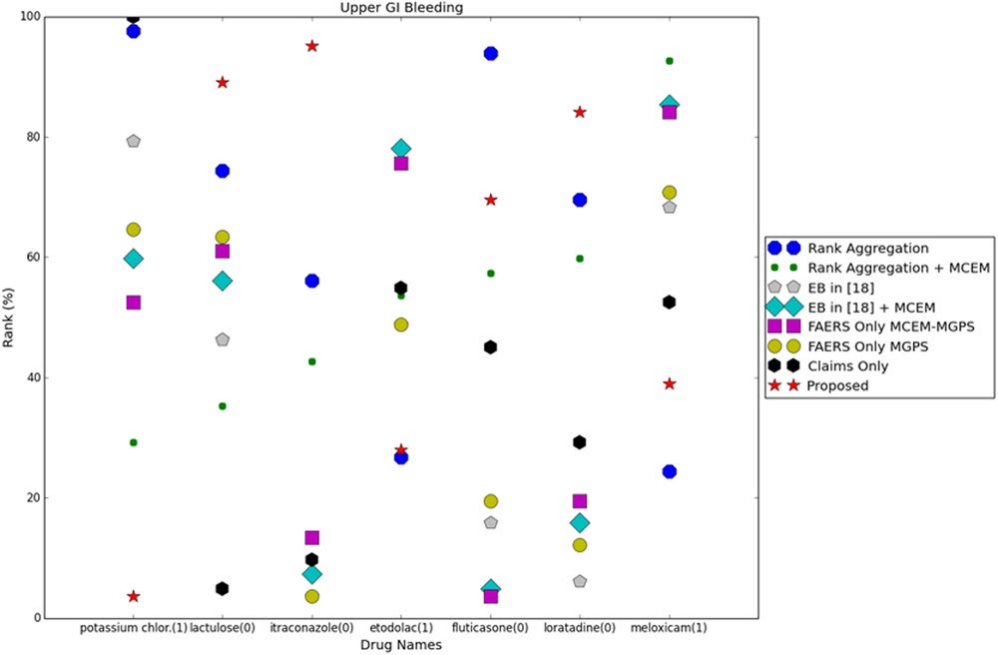
Comparison of signal strength for upper GI bleeding.

In Fig. 4, we compare the rankings of a few signals that indicate certain drugs would cause UGB. The signals in comparison are obtained using rank aggregation based on traditional MGPS, rank aggregation based on MCEM MGPS, EB with traditional MGPS, EB with MCEM MGPS, and the proposed method for all data are compared,. In addition, rankings of the traditional MGPS scores and MCEM MGPS scores for the combination of all the FAERS years only and combination of claims scores only based on EB in Harpez *et al.*^10^ are also included and named as FAERS Only MGPS, FAERS Only MCEM-MGPS, Claims Only.

On the horizontal axis drug names are given and 1 indicates a positive drug-ADR pair, whereas 0 indicates a negative pair. On the vertical axis ranking is given as percentile which was computed as $100\times \frac{{\rm{rank}}\,{\rm{of}}\,{\rm{the}}\,{\rm{pair}}}{{\rm{length}}\,{\rm{of}}\,{\rm{the}}\,{\rm{list}}}$ after sorting the obtained list in a descending order. For positive pairs, the proposed method is expected to enhance the signal and move the pair a higher rank compared to baseline methods. In the case of negative samples, the proposed method should not produce false positives in terms of rankings.

From Fig. 4, the proposed method behaved perfectly for Potassium Chloride, Lactulose, Itraconazole and Loratadine. For Melaxiam (positive sample) and for Fluticasone (negative sample), the proposed method gives higher and lower rankings compared to most of the baseline methods. If we look at the overall tendency, we observe that methods using MCEM MGPS can generate better rankings compared to traditional MGPS scores.

## Discussion

In this paper, we presented a new framework that detects improved drug safety signals from multiple and heterogeneous data sources via Monte Carlo Expectation-Maximization (MCEM) and signal combination. The MCEM procedure was designed to explicitly handle concomitant confounders in SRS data, where we propose a new sampling approach to generate more accurate SRS signals for each ADR through iteratively down-weighting their associations with irrelevant drugs in case reports. The signal combination step was designed to solve multiple data challenges of RWE, including data quality variance and bias, rare ADR and under-reporting issues. To alleviate these issue, we adopted Bayesian hierarchical model and proposed a new summary statistic such that SRS signals could be combined with signals derived from other observational health data allowing for related signals to borrow statistical support with adjustment of data reliability. We evaluated the proposed framework using real-world SRS and OHD data. Results demonstrated that the proposed framework outperformed state-of-the-art baselines and also detected many true signals that the baseline methods could not detect.

Future directions include (1) incorporate prior knowledge about drug-drug interaction (DDI) to filter out DDI confounders with modifications to the MCEM step. For example, for possible DDIs, we could draw from Binomial distribution to determine whether drug pairs are associated to a particular ADR, or (2) since different patient cohort could have different effects or reactions to drugs, it is necessary to identify cohort-specific signals. Given pre-defined cohorts, initial thoughts include for cohort-specific MGPS calculation, we could replace the Gamma-Poisson model by a Beta-Binomial model to identify cohort specific drug reaction signals.

In addition to future directions, we also identify some limitations in our study:

- One limitation is in the MCEM step, the iteration stopping is still based on a heuristic threshold determined by empirical studies. Therefore, the performance of the MCEM step is still sub-optimal. It could be further improved with better iteration stopping criteria.
- The second limitation is that although ADR instances induced by the combined use of two or more drugs are quite rare, it might be good to also handle these cases, i.e. the DDI cases. We did not handle DDI in this work, but proposed some possible approach as future studies.
- The third limitation is that the likelihood of drug-ADR associations might differ for different patient cohorts (e.g. demographic, gender, etc.) The proposed framework did not tackle this issue. We have also brainstormed some future directions to perform cohort-specific signal detection in our discussion of future work.

## Electronic supplementary material

Supplementary information
