# Supplementary material for: An MCEM Framework for Drug Safety Signal Detection and Combination from Heterogeneous Real World Evidence
Source: Sci Rep. 2018 Jan 29;8:1806. doi: 10.1038/s41598-018-19979-7 (PMC5789130; doi:10.1038/s41598-018-19979-7)
Supplement: Supplementary file 1 — Supplementary information [file 41598_2018_19979_MOESM1_ESM.pdf]

An MCEM Framework for Drug Safety Signal Detection and Combination from Heterogeneous Real World Evidence

Cao Xiao, Ying Li, Inci M. Baytas, Jiayu Zhou, and Fei Wang*

*few2001@med.cornell.edu

Supplementary Materials

A1: Monte-Carlo EM

The EM algorithm is an iterative method for the computation of the maximizer of some posterior density [17]. The basic idea behind the EM algorithm is to augment the observed data y by latent data z. The assumption is that given both y and z, it is straightforward to calculate and maximize the expectation of the augmented log-posterior $\log\big(p(\theta|z,y)\big)$. For the E-step, to obtain the maximizer of the observed posterior $p(\theta|y)$, one computes the expectation of $\log\big(p(\theta|z,y)\big)$ with respect to the conditional predictive distribution $p\big(z|y,\theta^{(i)}\big)$, where $\theta^{(i)}$ is the current approximation to the mode of the observed posterior. Here we call the maximizer Q function as in Eq. 1.

$$Q(\theta,\theta_0)=\int_Z \log\big(p(\theta|z,y)\big)p(z|\theta_0,y)\,dz \tag{1}$$

where Z denotes the sample space for the latent data z. The E-step is to compute the Q function and it follows the Maximization step (M-step). In the M-step, since we obtain the maximizer of this conditional expectation. The conditional predictive distribution is then updated using the new maximizer and the algorithm is iterated. By Jensen's inequality, if θ is chosen such that $Q(\theta,\theta_0)\geq Q(\theta_0,\theta_0)$, then $\log\big(p(\theta|z,y)\big)$ will be greater than or equal to $\log\big(p(\theta_0|y)\big)$. In this way, given the current approximation to the maximizer of the observed posterior $\theta^{(i)}$, we can maximize the Q function with respect to θ to obtain the update $\theta^{(i+1)}$. Due to the challenges in performing integration to Eq.2, a Monte Carlo sampling method was introduced to approximate the E-step. The Monte Carlo procedure is described as follows. Given the current approximation to the maximizer $\theta^{(i)}$, 1) we generate samples $z^{(1)},\ldots,\ z^{(m)}$ from the current approximation of the conditional predictive distribution $p\big(z|\theta^{(i)},y\big)$ and then 2) we update the current approximation to $Q_{i+1}\big(\theta,\theta^{(i)}\big)$ as a mixture of augmented log-posteriors of θ, mixed over the latent data patterns from 1). Then the Q function would be revised as Eq. 2:

$$Q_{i+1}(\theta,\theta_0)=\frac{1}{m}\sum_{j=1}^{m}\log\Big(p\big(\theta|z^{(j)},y\big)\Big) \tag{2}$$

Next, in the M-step, the conditional predictive distribution is then updated using the new maximizer as in Eq. 2. Here we iterate the process until the Q function value is not changing based on a heuristic threshold (e.g. $10^{-3}, 10^{-5}$). Note that to apply Eq. 2 in our case, we have z mapped

to the drug that is most likely to be the real cause of a query ADR in the particular record, and $z^{(1)}, \ldots, z^{(m)}$ are sampled from the drugs mentioned in the record.

Procedure A2

```
procedure MCEM-MTL PROCEDURE
    for Each source k in K do
        Initiate {φ_l} for each drug i, ADR j pair in y_ij
        while Convergence until Q_n values are no longer changing based on a heuristic
              threshold do
              for Each record do
                  for Each ADR j in the record do
                      Normalize {φ_l} to p_{i_1}, ...., p_{i_d}
                      Draw from Multinomial(p_{i_1}, ...., p_{i_d}) to assign drug i
                  end for
              end for
              for Each drug i, ADR j pair indexed as l do
                  Compute contingency table, in which n_00 only counts for reports with
                  assigned (i, j) pair.
                  Compute MGPS risk score y_lk and σ²_lk
              end for
        end while
    end for
    Compute y^(l) and σ²_l over K different sources.
    Signal Combination(y^(l), σ²_l ) ⇒ posterior {φ_l}
end procedure
```

Table A2: Distribution of OMOP test cases used in the evaluation from 2007 to 2014

**Year**		2007	2008	2009	2010	2011	2012	2013	2014
**AMI**	N^+	24	23	26	29	25	31	24	28
	N^-	31	28	46	40	44	46	43	44
**ARF**	N^+	19	18	20	20	20	19	18	17
	N^-	35	40	44	44	45	45	43	46
**ALI**	N^+	73	73	70	71	69	71	68	70
	N^-	23	23	28	27	24	26	28	25
**UGB**	N^+	21	21	22	23	23	23	21	22
	N^-	41	45	47	53	51	52	52	53
**Total**	N^+	137	135	138	143	137	144	131	137

	N-	130	136	165	164	164	169	166	168

N+: # of true positive cases; N-: # of true negative cases; AMI: acute myocardial infarction, ARF: acute renal failure, ALI: acute liver injury, UGB: upper gastrointestinal bleeding.

Table A3. Comparison of AUCs.

**SRS Dataset [based on adding0]**	**Avg. AUC Baseline**	**Avg. AUC MCEM MGPS**	**Avg. AUC MGPS***
FAERS all years	0.7346	**0.7532**	**0.7918**
Acute Myocardial Infarction (AMI)	0.5788	**0.6129**	**0.6447**
Acute Liver Injury (ALI)	0.7661	**0.7799**	**0.8484**
Acute Renal Failure (ARF)	0.8098	**0.8283**	**0.8321**
Upper GI bleeding (UGB)	0.7401	**0.7718**	**0.8064**

Table A4. Performance comparison performance for varying EB05 threshold.

**Threshold (EB05)**	**MCEM**				**MGPS**				**MGPS***			
	Sen	Spec	PPV	F1	Sen	Spec	PPV	F1	Sen	Spec	PPV	F1
1.0	0.32	0.94	0.84	0.47	0.34	0.93	0.81	**0.48**	0.30	0.98	0.93	0.45
1.5	0.24	0.96	0.85	**0.37**	0.16	0.98	0.89	0.27	0.21	0.99	0.94	0.34
2.0	0.18	0.98	0.87	**0.31**	0.08	1.00	0.94	0.15	0.16	0.99	0.95	0.27
2.5	0.15	0.98	0.88	**0.26**	0.04	1.00	0.96	0.08	0.13	1.00	0.96	0.22

Sen: sensitivity; Spec: specificity; PPV: positive predictive value (or precision); F1 is measured by the formula: 2*sensitivity*specificity/(sensitivity + specificity)

According to results in Table A4, the MCEM yielded higher sensitivity – retrieving higher number of true positive signals than the other methods at given thresholds, although it comprised the positive predictive value. Overall, the MCEM has the best F1 for the three of four threshold settings.
